# Essentiality of CTNNB1 in Malignant Transformation of Human Embryonic Stem Cells under Long-Term Suboptimal Conditions

**DOI:** 10.1155/2020/5823676

**Published:** 2020-09-24

**Authors:** Jie Liu, Sicong Zeng, Yang Wang, Juan Yu, Qi Ouyang, Liang Hu, Di Zhou, Ge Lin, Yi Sun

**Affiliations:** ^1^Institute of Reproductive & Stem Cell Engineering, School of Basic Medical Science, Central South University, Changsha 410001, China; ^2^Key Laboratory of Stem Cells and Reproductive Engineering, Ministry of Health, Changsha 410001, China; ^3^National Engineering and Research Center of Human Stem Cells, Changsha 410001, China

## Abstract

Human embryonic stem cells (hESCs) gradually accumulate abnormal karyotypes during long-term suboptimal culture, which hinder their application in regenerative medicine. Previous studies demonstrated that the activation of *CTNNB1* might be implicated in this process. Hence, the hESC line with stably silenced *CTNNB1* was established to further explore the role of *CTNNB1* in the malignant transformation of hESCs. It was shown to play a vital role in the maintenance of the physiological properties of stem cells, such as proliferation, migration, differentiation, and telomere regulation. Furthermore, the malignant transformation of hESCs was induced by continuous exposure to 0.001 *μ*g/ml mitomycin C (MMC). The results showed that *CTNNB1* and its target genes, including proto-oncogenes *CCND1* and *C-MYC*, were aberrantly upregulated in hESCs after MMC treatment. Moreover, the high expression of *CTNNB1* accelerated cell transition from G0/G1 phase to the S phase and stimulated the growth of cells containing breakage-fusion-bridge (BFB) cycles. Conversely, *CTNNB1* silencing inhibited these effects and triggered a survival crisis. The current data indicated that *CTNNB1* is intimately associated with the physiological properties of stem cells; however, the aberrant expression of *CTNNB1* is involved in the malignant transformation of hESCs, which might advance the process by facilitating telomere-related unstable cell proliferation. Thus, the aberrant CTNNB1 level might serve as a potential biomarker for detecting the malignant transformation of hESCs.

## 1. Introduction

Human embryonic stem cells (hESCs) are derived from the inner cell mass of blastocysts with the potential of unlimited self-renewal and pluripotent differentiation that makes it a candidate source of cells for regenerative medicine [1]. Numerous studies have demonstrated that the accumulated chromosomal aberrations in long-term suboptimal cultured hESCs are similar to those found in tumorigenesis and might interfere with the clinical application [2–4]. Consistently, our previous studies found that the human embryonic stem cell line, *ch*HES-3, gained increasing karyotypic abnormalities and progressed toward malignancy under long-term suboptimal culture *in vitro* [5, 6]. We also found that trace levels of mitomycin C (MMC), a DNA-damaging agent widely used for the preparation of feeder cells to support hESC growth, remained in the culture system which might be a major cause of these abnormalities [7]. Furthermore, we demonstrated that CTNNB1 was aberrantly upregulated in karyotypically aberrant hESCs under suboptimal culture conditions. However, under optimized culture conditions, hESCs with different passages maintained normal karyotype, and the expression of CTNNB1 did not display significant changes in karyotypically normal hESCs, thereby suggesting a link between *CTNNB1* and the malignant transformation of hESCs [6].

In humans, the Wnt/*β*-catenin signaling pathway has a pivotal function in early embryogenesis [8]. As an essential downstream mediator of this signaling, *β*-catenin (the protein encoded by the *CTNNB1* gene) is involved in the regulation and coordination of cell renewal, cell fate specification, and cell differentiation [9]. Deletion of *CTNNB1* results in a peri-implantation lethal phenotype in knockout mice, suggesting the vital role of *CTNNB1* during embryogenesis. The functional studies of *CTNNB1* in ESCs primarily focused on the regulatory characteristics of pluripotency and self-renewal [10]. However, the aberrant activation or mutation in *CTNNB1* is associated with several diseases as well as cancers, such as colon cancer, pancreatic cancer, lung cancer, ovarian cancer, hepatoblastoma, and thymoma [11, 12]. In recent years, the key functions of *CTNNB1* in tumorigenesis have been gradually revealed; it may facilitate the carcinogenic events by promoting cell proliferation and inhibiting cell apoptosis [13]. Our previous studies suggested that *CTNNB1* was also aberrantly upregulated in the malignant progression of hESCs, but the role of *CTNNB1* in this process remains unclear.

It is widely accepted that telomere is not only correlated to self-renewal ability and pluripotency of ESCs but also to the advanced invasive stage and poor prognosis of tumors [14–16]. Telomeres are composed of tandem repeats of the (TTAGGG)_n_ DNA sequence and associated protein complexes that exert a protective effect on the chromosome ends. In normal somatic cells, the telomeres are shortened in each round of cellular division [17]. After telomere degradation reaches a critical level, uncapped telomeres induce replicative senescence or apoptosis to maintain genomic integrity [18]. Intriguingly, telomere maintenance is a key feature of human malignant cells and is required for the infinite proliferation and maintenance of other cancer hallmarks [19]. Our previous studies indicated that both abnormal shortening and elongation are associated with the tumorigenesis of hESCs, and the telomere dysfunction is responsible for complex chromosomal aberrations [20]. Accumulating evidence suggested that telomeres are crucial for cellular homeostasis and that telomere dysfunction can initiate genome instability and potentially trigger events that culminate in cancer [21]. As successive cell divisions occur, telomere dysfunction accumulates chromosomal instability and encourages the fusion of chromosome ends [22]. This break-fusion-bridge (BFB) event results in substantial chromosomal rearrangements, especially translocations and aneuploidy [23]. These processes promote malignant cellular transformation via stochastic inactivation of tumor suppressor genes and the activation of oncogenes [24].

Although these studies indicated that *CTNNB1* and telomere are involved in the maintenance of stem cell characteristics and genomic stability, their correlation with the malignant transformation of hESCs remains to be elucidated [25, 26]. In this study, we established a *CTNNB1*-deficient human embryonic stem cell line by short-hairpin RNA (shRNA) lentivirus to investigate the role of *CTNNB1* in maintaining the stem cell physiological properties and malignant transformation of hESCs. The current data revealed that *CTNNB1* deficiency not only suppresses the capacity of proliferation, migration, and differentiation of hESCs but also shortens the telomere length by reducing the telomerase activity. Further investigation indicated that the overexpression of *CTNNB1* and its target genes, including proto-oncogenes *CCND1* and *C-MYC*, was accompanied by severe BFB events, which drives the complex chromosomal rearrangements and consequent malignant transformation of stem cells. Moreover, telomere regulation is intimately related to this process. These results provided new insights into the complex biology of the malignant transformation of hESCs and suggested that CTNNB1 might be a potential biomarker for this process.

## 2. Materials and Methods

### 2.1. Cell Culture

The *ch*HES-3 cells with normal karyotype (Normal), karyotypically aberrant *ch*HES-3 cells with simple duplication karyotype (SIMP) and complex karyotype (COMP), and all the other hESC lines were established and cultured in our laboratory as previously reported [27]. Briefly, cells were grown on ICR MEF (Harlan Laboratories, USA) feeders inactivated by 10 *μ*g/ml mitomycin C (Sigma-Aldrich, USA) and cultured in serum-free DFSR medium, containing knockout DMEM/F12 (Gibco-BRL, USA) supplemented with 15% serum replacement (Gibco-BRL, USA), 0.1 mM *β*-mercaptoethanol (Sigma-Aldrich, USA), 1% nonessential amino acids (Gibco-BRL, USA), 2 mM L-glutamine (Gibco-BRL, USA), and 4 ng/ml human recombinant basic fibroblast growth factor (Gibco-BRL, USA). The cells were passaged by mechanical dissection every 6 days. The human embryonal carcinoma cell (hECCs) line NTERA-2 cl.D1 (EC) was cultured on matrigel-coated plates under conditions described previously for this cell line [28, 29]. The derivation experiment was approved and guided by the ethical committee of CITIC-Xiangya Reproductive & Genetic Hospital (ethical permission number 2001-01).

### 2.2. ITRAQ Proteomics Analysis

ITRAQ proteomics analysis among Normal, SIMP, and COMP *ch*HES-3 cells, as well as NTERA-2(EC) cells, was carried out as previously reported [29]. Briefly, cells were washed three times with ice-cold PBS collected by centrifugation at 1000 rpm and were suspended in 200 *μ*l of lysis buffer (7 M urea, 1 mg/ml DNase I, 1 mM Na3VO4, and 1 mM PMSF) at 4°C. The cell lysate was subjected to intermittent sonication using a Vibra Cell™ high-intensity ultrasonic processor (Jencon, UK). The protein concentration of cleared lysates was determined by 2-D Quantification kit (Amersham Biosciences, Sweden) according to the manufacturer's instructions.

Approximately 100 *μ*g of proteins were reduced with 5 mM tris-carboxyethyl phosphine hydrochloride (TCEP) for 60 min at 37°C, alkylated with 10 mM methylethanethiosulfonate (MMTS) for 20 min at room temperature (RT), and then diluted 10 times with deionized water prior to the digestion with 2 *μ*l of 0.25 *μ*g/*μ*l sequencing grade trypsin (Promega, USA) overnight at 37°C. Peptides generated were labeled with iTRAQ reagents according to the manufacturer's protocol (Applied Biosystems/MDS SCIEX, USA). The samples were labeled with the respective tags as follows: *ch*HES-3 cell subsets including Normal, SIMP, and COMP *ch*HES-3 cells were labeled with reporter tags 114, 115, and 116, respectively. And hECCs were labeled with reporter tags 117.

ITRAQ-labeled tryptic peptide samples were fractionated by Isoelectric Focusing (IEF) on immobilized pH gradient (pH 3-10, 18 cm long, Amersham Biosciences, Sweden). These fractions were lyophilized in a vacuum concentrator and subjected to C-18 cleanup using a C18 Discovery DSC-18 SPE column (100 mg capacity, Supelco, USA). The cleaned fractions were then lyophilized again and stored at -20°C prior to mass spectrometric analysis. Ten microliters of sample was injected into the nano-LC−ESI−MS/MS system for each analysis. Mass spectrometry was performed using a QStar Elite Hybrid ESI Quadrupole time-of-flight tandem mass spectrometer (ESI-Q-TOF-MS/MS, Applied Biosystems/MDS-Sciex, Canada) coupled to an online capillary liquid chromatography system (Dionex, The Netherlands). The mass spectrometer was set to perform data acquisition in the positive ion mode, with a selected mass range of 300–1800 m/z. The time of summation of MS/MS events was set to be 2 s. This refers to the amount of time allowed for the machine to accumulate MS/MS events before switching back to MS scan. The two most abundant charged peptides above a 20-count threshold were selected for MS/MS and dynamically excluded for 30 s with ±50 mDa mass tolerance. Protein identification and quantification for iTRAQ samples were carried out using the ProteinPilot software (version 2.0; Applied Biosystems/MDS-Sciex, USA). The database search was performed by setting cysteine modification by MMTS as a fixed modification. Other parameters include mass tolerance of up to 0.2 Da, maximum of one missed cleavage of trypsin, oxidation of methionine, N-terminal iTRAQ labeling, and iTRAQ labeled-lysine. Relative quantification of proteins in the case of iTRAQ is performed on the MS/MS scans and is the ratio of the areas under the peaks at 114, 115, 116, and 117 Da, which were the masses of the tags that correspond to the iTRAQ reagents used to label the samples. The statistical calculation for iTRAQ-based detection and relative quantification was performed using the Paragon Algorithm 19 embedded within the ProteinPilot software. Following data analysis by the ProteinPilot software, the protein summary results were exported into an Excel sheet and manually inspected and processed. Briefly, for protein identification and quantitative analysis, 95% confidence was used. Protein identification must be based on at least two unique peptides, and the *p* values for the relative quantification by iTRAQ must be <0.05. Protein hits that do not satisfy these criteria are removed.

### 2.3. Stable Transfection

*CTNNB1* shRNAs plasmid, pLKO.1-puro-shCTNNB1 (pLKO.1 puro shRNA beta-catenin, Plasmid #18803) were from Addgene, with empty vector (pLKO.1 puro, Plasmid, #8453) as control. Viral particles were packaged in virus packaging cell line 293FT and collected according to the manufacturer's instructions, then added to mTeSR™1 medium (StemCell Technologies, Canada) with 8 ng/ml polybrene and incubated overnight; the second infection was performed on the next day. After infection, puromycin resistance hESC clones were selected in the medium containing 1 *μ*g/ml puromycin (Sigma-Aldrich, USA) for two weeks. The positive clones were picked and expanded to establish cell lines, and stable transfection cell lines were determined by the Western blot analysis.

### 2.4. Western Blot Analysis

The cells were harvested from dishes, washed twice with cold PBS, and lysed in RIPA lysis buffer (Beyotime, China) for 60 minutes on ice, followed by centrifuging at 11,000 × g for 15 min at 4°C to remove cell debris. Then, the supernatant was collected and the protein concentrations were determined by BCA protein assay (Sigma-Aldrich, USA). After the addition of 2× loading buffer, eighty micrograms of lysates were boiled at 95°C for 5 min and were separated on 10% or 12% SDS-PAGE gels. Proteins were subsequently electrotransferred to PVDF membranes (Millipore, Germany). After blocking with 5% nonfat dry milk in TBS-T containing 0.1% Tween-20 for 2 h at room temperature, the membranes were probed with anti-CTNNB1 (88 kDa) or anti-*β*-Actin (43 kDa) diluted 1 : 1000-1 : 2000 overnight at 4°C, followed by incubation in a 1 : 2000 dilution of secondary antibodies conjugated to horseradish peroxidase for 1 h at room temperature. Protein bands were detected using the ECL detection system, followed by exposure on Hyperfilm (Amersham Biosciences, Sweden). All the western immunoblots were performed at least three times. In each experiment, membranes were also probed with anti-*β*-Actin antibody to correct for differences in protein loading. Image analysis system (Image J for windows) has been applied to analyze the strap of Western blot.

### 2.5. Immunofluorescence Staining and Alkaline Phosphatase Activity Assay

Antibodies used for immunofluorescence staining and Western blot analysis are summarized in Supplementary Table 1. CTNNB1, ESCs-specific surface markers, and three germ layers markers were tested by immunostaining. The ESCs-specific surface markers were consisted of OCT-4, TRA-1-60, TRA-1-81, SSEA-4, SSEA-3, SSEA-1, and NANOG. Three germ layer markers consisted of *β*-tubulin (ectoderm), SMA (mesoderm), and AFP (endoderm). Cells were fixed in 4% paraformaldehyde for 15 min, permeabilized with 0.2% Triton X-100 for 10 min, and blocked in 4% goat serum in PBS for 1 h. Cells were incubated with primary antibody overnight at 4°C. Then, the cells were stained using Alexa Fluor (Invitrogen, USA) secondary antibody for 1 h. Nuclei were counterstained with 4′,6-diamidino-2-phenylindole (DAPI, KPL, USA). Alkaline phosphatase activity was detected according to the protocol of the Fast-Red Substrate (Zymed Laboratories, USA).

### 2.6. Reverse Transcription-PCR (RT-PCR)

Total RNA was extracted using TRIzol reagents (Gibco-BRL, USA) according to the manufacturer's instructions. Two micrograms of RNA per sample was reverse-transcribed into first-strand cDNA by using the Transcriptor First Strand cDNA Synthesis Kit (Roche Diagnostics, Germany). For RT-PCR assay, the thermal cycling conditions were as follows: initial denaturation at 95°C for 2 min, followed by 35 cycles of amplification (95°C for 30 s, 54–64°C for 30 s, and 72°C for 30 s) and a final extension at 72°C for 5 min. The PCR products were separated on a 1.5% agarose gel by electrophoresis and visualized on a UV transilluminator. Primer pairs are shown in Supplementary Table 2. Image analysis system (Image J for windows) has been applied to analyze the strap of RT-PCR.

### 2.7. Real-Time Quantitative PCR (RT-qPCR)

RT-qPCR amplifications were performed on the Roche LightCycler system (Roche Diagnostics, Germany) with SYBR Green I dye. The cDNA was submitted to real-time PCR using the following primer pairs in Supplementary Table 2. The amplification conditions included an initial denaturation step at 95°C for 5 min followed by 40 cycles of 95°C for 30 s, 56°C for 30 s, and 72°C for 30 s. After each run, the cycle threshold (CT) values were provided from real-time PCR instrumentation by the LightCycler software. The analysis of the relative gene expression was performed by using the 2^-∆∆CT^ method described by Livak and Schmittgen [30]. Evaluation of 2^-∆∆*C*T^ indicates the fold change in gene expression relative to the internal standard gene *GAPDH* and takes into account the standard deviation. Individual CT values were based on three separate measurements. The specificity of the PCR amplification was directly verified by the melt curve analysis of the final products in the iCycler.

### 2.8. Karyotype Analysis

The hESCs were cultured in mTeSR™1 medium (StemCell Technologies, Canada) for 3-5 days and then treated with a 0.06 *μ*g/ml KaryoMAX® Colcemid™ solution (Gibco-BRL, USA) for 2.5 h. After washing with PBS three times, the cells were incubated in Accutase (Millipore, USA) at 37°C for 2-3 min and harvested using standard procedures, followed by standard G-banding for karyotyping. At least 50 metaphase spreads were examined for each sample using an Olympus fluorescence microscope BX51 (Olympus, Japan) with LUCIA KARYOTYPE software (Lucia, Czech Republic).

### 2.9. Embryoid Bodies Formation

Spontaneously *in vitro* differentiation was conducted through embryoid body (EB) formation. hESC colonies were mechanically dissociated into small clumps and detached to grow as aggregates in suspension for 7 days to form embryoid bodies in DFSR medium without bFGF. The embryoid bodies were then transferred to a gelatin-coated six-well plate for adherent culture for 3-8 days in the same medium.

### 2.10. Analysis of Cell Growth *in Vitro*

For cell proliferation and cytotoxicity assay, the aliquots of cell suspension containing 500 cells in 100 *μ*l of medium were transferred into the individual well of 96-well tissue culture plates and were grown for 7 days. Every 24 h, 10 *μ*l of Cell Counting Kit-8 (BBI Life Sciences Co., China) was added to wells, and the medium was removed after 4 h of incubation. The absorbance of each well was read with a Bio-Tek Instruments EL310 Microplate Autoreader (BioTek Instruments, USA) at 450 nm. The percentage of cell growth was calculated by the comparison of the A450 readings versus the first day of absorbance. Each experiment was performed at least three times in triplicate.

For the analysis of the cell proliferation, 1 × 10^7^ ES cells were cultured for 12 h in 20 ml EdU (5-ethynyl-20-deoxyuridine) medium and then harvested and stained by the Click-iTTM EdU Alexa Fluor 488 Cell Proliferation Assay Kit (Invitrogen, USA) in accordance with the manufacturer's protocol. Fluorescence data were collected using a FACS Calibur (Becton Dickinson, USA).

### 2.11. Cell Cycle Analysis by Flow Cytometry

For cell cycle analysis, 1 × 10^6^ cells were harvested, washed twice with cold PBS buffer, and fixed with 70% cold ethanol. After incubation at 4°C overnight, cells were washed with PBS, resuspended in FACS buffer containing RNase A (0.2 *μ*g/ml) and propidium iodide (20 *μ*g/ml, Sigma-Aldrich, USA), and incubated at 37°C for 30 min. The stained cells were analyzed on a FACScan flow cytometer (Becton Dickinson, USA) with excitation at 488 nm and the emission recorded 675 nm long pass (FL4, mitoxantrone) filters, and the data were analyzed by the ModFIT/LT software.

### 2.12. Wound Healing Assay

Cell migration was detected by wound healing assay, which is a measure of the speed of the collective motion of the cells. Plate 5 × 10^5^ cells in a plastic-bottomed 6-well dish. Mitomycin C (10 *μ*g/ml) was incubated for 2 h to arrest mitosis when the cells were fused to 90%. To simulate wounding, scratch a confluent monolayer with a pipette tip for a gap size of 0.5 mm, rinse the wound with DPBS to remove debris, and replace it with a serum-free medium for continuous culture. Record the wound healing images at 0 h, 18 h until the gap is approximately half-closed. Image analysis system (Image J for windows) has been applied to analyze the scratch area at each time point and calculate the cell mobility.

### 2.13. Telomerase Activity

Quantitative measurement of telomerase activity was performed using the TRAPeze Telomerase Detection Kit (Millipore, USA) according to the manufacturer's instruction. In brief, after the addition of 100-200 *μ*l CHAPS buffer, the cells were lysed on ice for 30 min and then centrifuged at 12,000 rpm for 20 min at 4°C. The supernatant was collected, and the protein concentrations were determined by standard procedures (BCA protein assay, Sigma-Aldrich, USA). In each reaction, a volume of 0.33 *μ*g protein equivalent was added to a 48 *μ*l reaction solution consisting of TRAP buffer, dNTP Mix, TS primer, RP primer mix, and 2 U Hotstart Taq polymerase (Qiagen, USA). After incubated at RT for 30 min and stop at 80°C for 10 min, telomerase activity was determined by real-time quantification of the PCR products using a Roche Lightcycler II.

### 2.14. Telomere Length Quantitative FISH

For Q-FISH analyses on metaphase, cells were grown for 3–4 days after passage and stalled at metaphase by incubation with 0.1 mg/l colcemid for 1.5 h. After that, cells were collected, treated in 0.56% KCl buffer for 10 min, and fixed 3 times in fixation buffer (methanol to acetic acid, 3 : 1). Metaphase spreads were prepared by dropping fixed cells onto cleared glass slides. The Q-FISH procedure was performed as previously described [31]. Cell line LY-S and LY-R were employed to generate the calibration curve in every experiment. Fluorescence images were captured on fixed exposure timing and insensitivity, and telomere length was determined by measuring the telomere fluorescence intensity, which was evaluated by using the Tel-TELO program.

### 2.15. Break-Fusion-Bridge Detection

For anaphase bridge scoring, cells were cultured on coverslips to approximately 80% confluence and fixed with 4% paraformaldehyde (PFA, Sigma-Aldrich, USA) at room temperature (RT), rinsed twice in PBS, allowed to dry, and counterstained with DAPI. The number of anaphase bridges per total anaphase figures was then evaluated.

### 2.16. Statistical Analysis

All observations were confirmed by at least three independent experiments. Analysis of variance (ANOVA) followed by a Fisher's protected least significant difference (LSD) test was used to analyze all experiments, and the results were expressed as mean ± standard deviation (SD). These analyses were done using the Statistical Package for Social Science software (SPSS for Windows, version 10.0). A value (*p* < 0.05) was considered statistically significant.

## 3. Results

### 3.1. Quantitative Proteomics Showed Upregulated Expression of CTNNB1 Proteins in a Malignant Transformed Stem Cell Line

**Figure 1:**
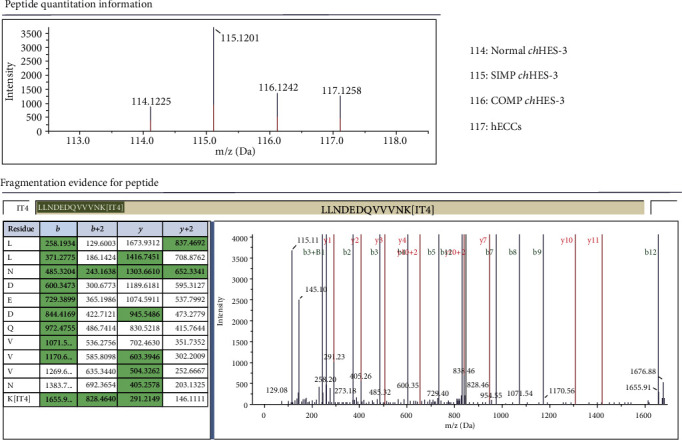
CTNNB1 expression is upregulated during the malignant transformation of hESCs. MS/MS spectra of iTRAQ-labeled peptides from CTNNB1. (a) The relative peak heights of iTRAQ labels. (b) A representative tandem mass spectrum of a peptide in each protein. Reporter ions in (a) demonstrate examples of equal, low, and high protein levels in normal and aberrant *ch*HES-3 cells. Normal, SIMP, COMP, and NTERA-2 cells are labeled as 114, 115, 116, and 117, respectively. The *x*-axis of a mass spectrum represents the correlation between the mass of a given ion and the number of elementary charges. This is represented as the IUPAC standard m/z to denote the quantity generated by dividing the mass of an ion by the unified atomic mass unit and its charge number (positive absolute value). Data are represented in Dalton (Da) as the unit of mass. The *y*-axis of a mass spectrum represents the signal intensity of ions.

Our previous studies reported that the *ch*HES-3 cell line underwent karyotypic changes from simple to complex and experienced dysregulation of self-renewal pathway and dysfunction of related oncogenes during long-term suboptimal culture, leading to malignant transformation (Yang et al. [5]). Hence, *ch*HES-3 is a specific cell model for the investigation of the mechanism underlying stem cell malignant transformation. Significantly, the intracellular Wnt signaling pathway was preferentially activated in malignant transformed *ch*HES-3 cells, especially the expression of *CTNNB1* was aberrantly upregulated, which might be associated with this process [6]. To further investigate the differential expression of CTNNB1 during the *ch*HES-3 malignant transformation, Normal (*ch*HES-3 cells retained a normal karyotype), SIMP (*ch*HES-3 with a simple duplication karyotype), and COMP (*ch*HES-3 with the accumulation of more chromosomal abnormalities) *ch*HES-3, as well as human embryonal carcinoma cell (hECCs) line NTERA-2 cl.D1 (EC) were analyzed by quantitative proteomics. Four unique reporter ions (m/z = 114‐117) were used to label the Normal, SIMP, COMP *ch*HES-3 cells, and EC cells, respectively. Consistent with a previous study [6], the MS/MS spectra and iTRAQ ratios of the peptides from *β*-catenin showed a high expression of CTNNB1 in karyotypically aberrant *ch*HES-3 (Figure 1).

### 3.2. Reduced Differentiation Capacity in CTNNB1-Depleted Stem Cells

**Figure 2:**
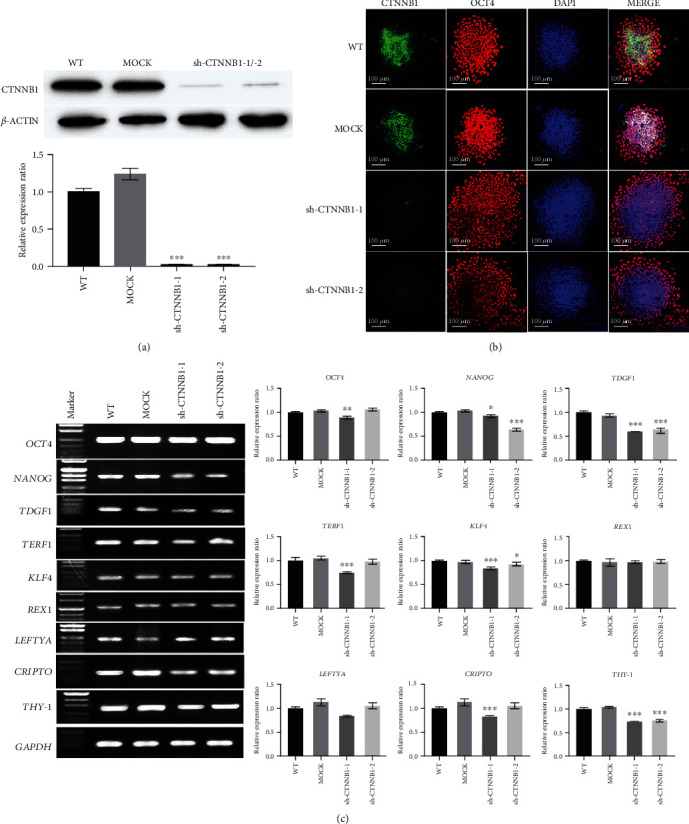
shRNA induces the downregulation of CTNNB1 expression in hESCs. (a) Western blot analysis of CTNNB1 protein in the shCTNNB1 group, empty vector group (MOCK), and uninfected control group (WT) hESCs. (b) Immunofluorescence assay of CTNNB1 and OCT4 in shRNAs, MOCK, and WT hESCs (scale bar = 100 *μ*m). (c) Semiquantitative RT-PCR validation of mRNA expression levels of pluripotency genes in shCTNNB1, MOCK, and WT hESCs. Error bars denote mean ± SD from three independent experiments (*n* = 3, ^∗^*p* < 0.05, ^∗∗^*p* < 0.01, ^∗∗∗^*p* < 0.001, relative to WT hESCs).

**Figure 3:**
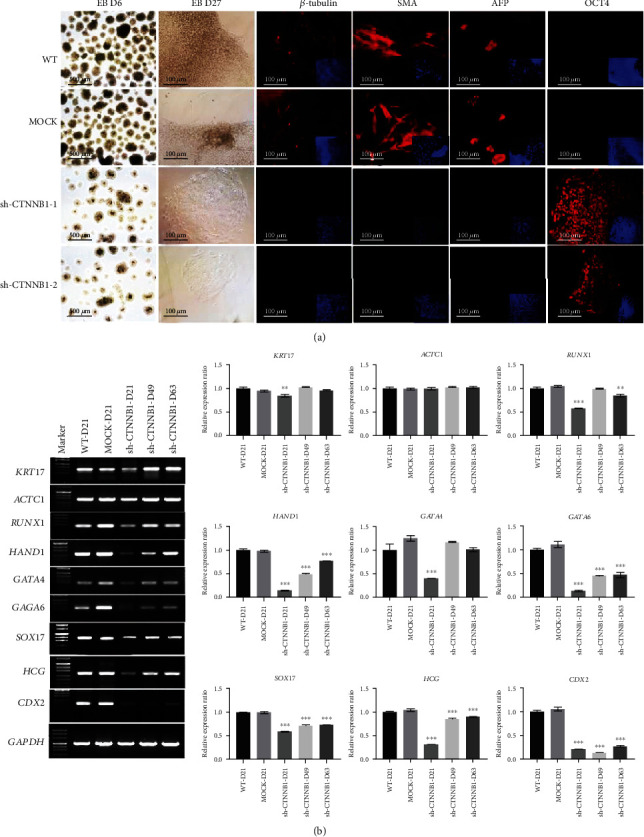
Suppression of multilineage differentiation capability in shCTNNB1 hESCs. (a) Immunofluorescence staining shows *β*-tubulin, SMA, and AFP triploblastic markers in 21-42 days differentiated hESCs (scale bar = 100 − 500 *μ*m). (b) Semiquantitative RT-PCR validation of mRNA expression levels of the three germ layers in 21-42 days differentiated hESCs. The error bars denote mean ± SD from three independent experiments (*n* = 3, ^∗^*p* < 0.05, ^∗∗^*p* < 0.01, ^∗∗∗^*p* < 0.001, relative to WT hESCs).

*CTNNB1-*deficient hESC colonies were established by shRNA infection, and the CTNNB1 expression and stem cell characteristics were explored after interference (Figures 2 and 3 and Supplemental Figure 2). The silencing efficiency of *CTNNB1* was measured by Western blot and immunofluorescence assays. The results showed that compared to the wild-type (WT) and empty vector group (MOCK), the expression of CTNNB1 was remarkably decreased in the shCTNNB1 group, while no significant difference was detected between the MOCK and WT groups (Figures 2(a) and 2(b)). The G-band analysis after long-term culture showed that hESCs after *CTNNB1* interference retained a normal karyotype (Supplemental Figure 1(b)). Immunofluorescence and RT-PCR results indicated that the downregulation of the *CTNNB1* level decreased the expression of intrinsic pluripotency factors, including *OCT4*, *NANOG*, *TDGF1*, *TERF1*, *KLF4*, *REX1*, *CRIPTO*, and *THY-1* (Figures 2(b) and 2(c)).

Interestingly, shCTNNB1-hESCs formed smaller and fewer embryonic bodies (EBs) that grew slowly 7 days after replating into the dishes, which was opposite to that of the control and WT groups under identical conditions (Figure 3(a)). In addition, immunofluorescence using antibodies against *β*-tubulin (ectoderm), SMA (mesoderm), and AFP (endoderm) confirmed that both the WT and MOCK groups are differentiated into three germ layers, but no positive signal was recorded in the shCTNNB1 group. Surprisingly, the majority of the shCTNNB1-hESCs persistently showed strong OCT4-positive signal even after 27 days of culture in the differentiation medium (Figure 3(a)). RT-PCR analysis showed a significantly lower expression of the three germ layer markers such as *KRT17*, *ACTC1*, *RUNX1*, *HAND1*, *GATA4*, *GATA6*, *SOX17*, *HCG*, and *CDX2* in shCTNNB1-hESCs as compared to WT and MOCK groups (Figure 3(b)), suggesting a conspicuous inhibition of differentiation in hESCs after *CTNNB1* interference. Taken together, these results indicated that continuous expression of pluripotency markers is exhibited in *CTNNB1*-interfered hESCs, and the expression of differentiation markers is suppressed.

### 3.3. Suppressed Proliferation and Migration Capacity in CTNNB1-Depleted Stem Cells

**Figure 4:**
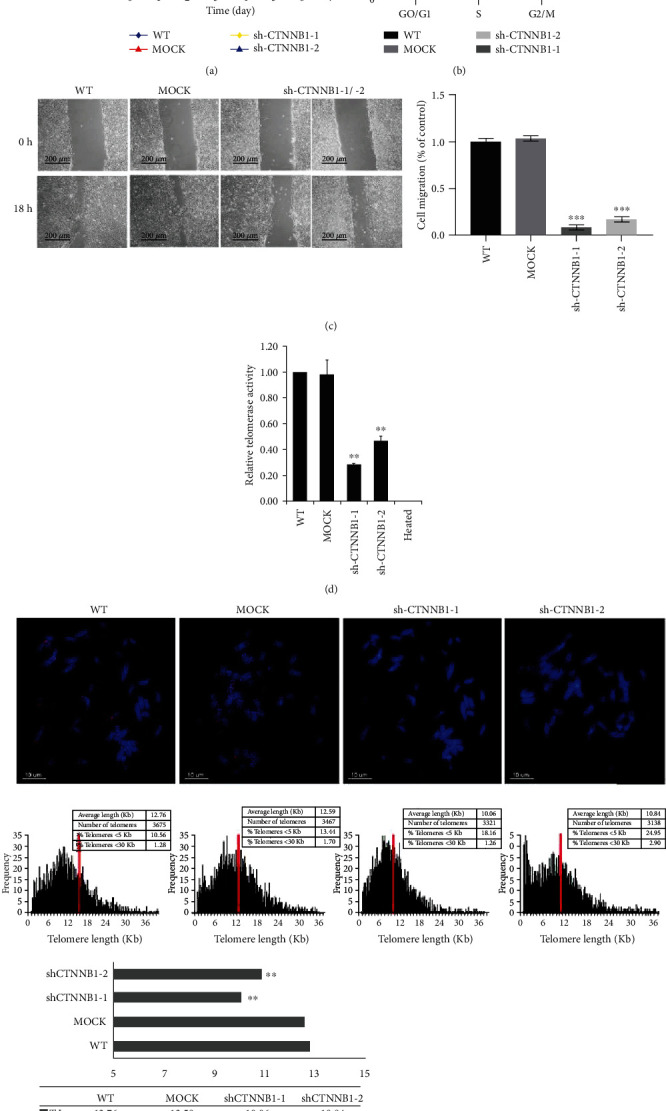
shCTNNB1 hESCs exhibit low cell proliferation and decreased telomere length as compared to WT cells. (a) Cell growth curves of shCTNNB1, MOCK, and WT hESCs. The data are represented as an average of two independent experiments (*n* = 2). (b) Cell cycle analysis of shCTNNB1, MOCK, and WT hESCs. (c) Wound-healing assay. Wounds were created by manual scraping the cell clones, and images were captured as the reference point. The image of the healing state was acquired after 18 h (scale bar = 200 *μ*m). (d) Decreased telomerase activity in shCTNNB1 cells. Heated HSF and WT cell lysates were used as negative controls. (e) Telomere length in shCTNNB1 hESCs. Metaphase cells from the indicated groups were analyzed by the quantitative telomere-FISH assay. Strand-specific telomeric DNA probes (red) show telomere repeats; the nuclei were stained with DAPI (blue). Telomere length is represented in arbitrary units of fluorescence (a.u.) (scale bar = 10 *μ*m). The histograms show the frequency of different telomere lengths in *CTNNB1*-silenced hESCs. The results are represented as an average of two or more independent experiments. Error bars indicate mean ± SD from three independent experiments (*n* = 3, ^∗^*p* < 0.05, ^∗∗^*p* < 0.01, relative to WT hESCs).

To further evaluate the role of *CTNNB1* in hESCs, we investigated the cell proliferation and migration capabilities of shCTNNB1-hESCs. The results indicated that in comparison with the WT and MOCK groups, the interference of *CTNNB1* distinctly delayed the growth rate of hESCs (Figure 4(a) and Supplemental Figure 1(a)). The cell cycle assay was used to further investigate the effect of *CTNNB1* on cell cycle distribution, the percentage of cells in the G0/G1 phase was increased markedly after *CTNNB1* silencing, while a significant decrease was observed in the S phase as compared to the control groups (Figure 4(b)). Moreover, the wound healing assay in shCTNNB1-hESCs showed a significantly slower healing process as compared to the control group (Figure 4(c)). These data indicated that knocking down *CTNNB1* in hESCs decreases the rate of cell proliferation, causes G0/G1 cell cycle phase arrest, and lowers the rate of cell migration, thereby implying its function in supporting the cell growth and migration.

### 3.4. Decreased Telomerase Activity and Shortened Telomere Length in CTNNB1-Depleted Stem Cells

In hESCs, telomere regulation is one of the crucial mechanisms that maintain stem cell characteristics. Also, Wnt/*β*-catenin signaling is reported to play a major role in regulating telomerase reverse transcriptase (Tert) expression and telomere length in mESCs [32]. Herein, we compared the telomerase activity and telomere length in *CTNNB1*-depleted hESCs, WT, and MOCK groups. The TRAPeze Telomerase Detection Kit was used to evaluate the telomerase activity, and lower telomerase activity was detected in *CTNNB1*-depleted hESCs than in the WT and MOCK groups (Figure 4(d)). Furthermore, a short telomere length (about 2.2 Kb shortening) was detected in *CTNNB1*-depleted hESCs by quantitative fluorescence in situ hybridization (Q-FISH) (Figure 4(e)). These results suggested a positive role of *CTNNB1* in the maintenance of telomeres of hESCs.

### 3.5. CTNNB1 Is Also Required for the Survival and Proliferation of Cells with Genomic Instability

**Figure 5:**
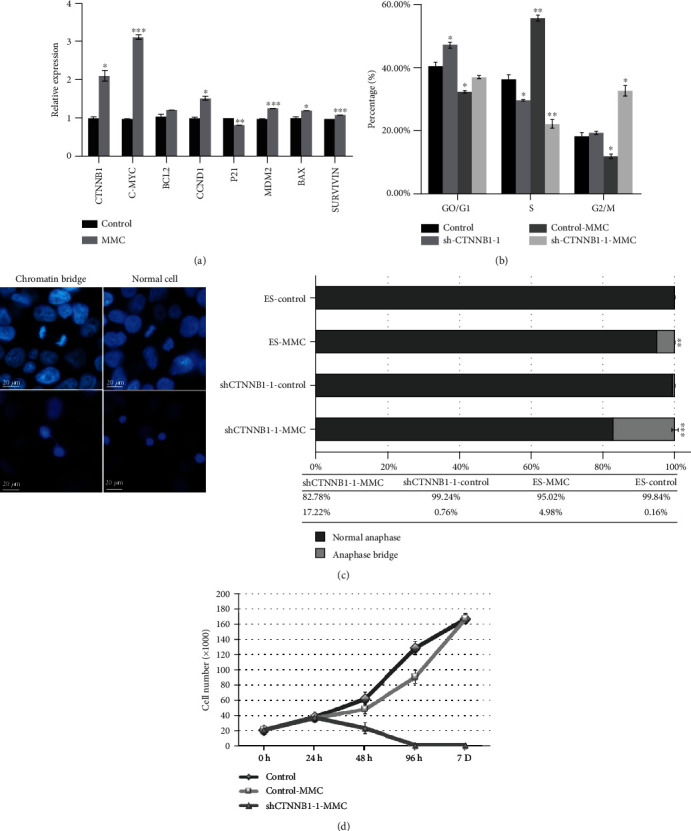
MMC-induced malignant transformation and BFB in hESCs. (a) qRT-PCR analysis of genes expressed in 0.001 *μ*g/ml MMC-treated cells after 10 passages. (b) Cell cycle analysis of MMC-treated cells. (c) Chromatin bridge analysis was performed in hESCs after 0.001 *μ*g/ml MMC treatment for 48 h. Nuclei were stained with DAPI (blue) (scale bar = 20 *μ*m). The total number of cells in anaphase and anaphase bridge was counted. (d) Cell growth curves of MMC-treated cells. The error bars represent mean ± SD from three independent experiments (*n* = 3, ^∗^*p* < 0.05, ^∗∗^*p* < 0.01, ^∗∗∗^*p* < 0.001, relative to control hESCs).

To further understand the role of *CTNNB1* during the malignant transformation of hESCs, cells were continuously exposed to 0.001 *μ*g/ml MMC for 10 passages to activate the malignant transformation of hESCs. RT-qPCR revealed that the expression level of *CTNNB1* and its target genes, including proto-oncogenes *CCND1*, *C-MY*C, *MDM2*, and *BCL-2*, was upregulated, while that of the apoptosis-related molecule, *P21*, was downregulated (Figure 5(a)). Next, we treated *CTNNB1*-silenced hESCs with MMC in order to evaluate the cellular response after *CTNNB1* knockdown. Cell cycle analysis demonstrated that the overexpression of *CTNNB1* was accompanied by a decreased population in the G0/G1 phase and a significantly increased population in the S phase. Conversely, the population in the G2/M phase in shCTNNB1-hESCs treated with MMC was increased, and that in the S phase was decreased (Figure 5(b)). Furthermore, severe BFB events were triggered in shCTNNB1-hESCs as compared to the control groups after MMC treatment (Figure 5(c)). The control groups showed superior proliferation ability as compared to shCTNNB1-hESCs and died within one week after the MMC treatment (Figure 5(d)).

## 4. Discussion

Although hESCs are promising seed cells for regenerative medicine, the accumulated chromosomal abnormalities during prolonged culture is a major bottleneck that hinders its clinical application. Previous studies have reported that malignantly transformed genetically unstable hESCs harbor the classical stem cell surface markers similar to normal hESCs, and therefore, it is difficult to selectively remove unhealthy hESCs from healthy cultures [33]. Our previous studies demonstrated that the overexpression of *CTNNB1* was accompanied by increased karyotype complexity during the malignant transformation, indicating a putative correlation between *CTNNB1* and the malignant transformation of hESCs [5, 6]. Therefore, the present study aimed to investigate the effects of *CTNNB1* in the malignant transformation of hESCs, which is conducive to the safety assessment of the clinical application of hESCs.

In mammals, *CTNNB1* controls cell proliferation and cell fate decisions before and after birth and plays a crucial role at multiple stages of embryonic development [34]. The current results also indicated that in normal physiological conditions, *CTNNB1* played an essential role in maintaining cell proliferation, migration, and differentiation capacities of hESCs; it also participates in cell cycle and telomere regulation. Since the Wnt/*β*-catenin signaling pathway is one of the integral structural components of the crucial nuclear effector, the hyperactive Wnt/*β*-catenin signaling was involved in a wide variety of diseases, and hence, the imbalance and dysregulated overexpression of *CTNNB1* is related to several human cancers [12, 35]. Consistently, our previous results suggested that *CTNNB1* was aberrantly upregulated accompanied by chromosomal aberrations in transformed hESCs [5, 6]. These findings indicated that *CTNNB1* plays an essential role in the maintenance of the physiological functions of hESCs; however, the dysregulation of *CTNNB1* might be related to the malignant transformation of hESCs.

Previously, we reported that the trace level of MMC, which is an adverse factor for malignant transformation of hESCs, causes karyotype abnormalities and carcinogenesis in long-term cultured hESCs [7]. Thus, we optimized our culture conditions by using *γ*-inactivated feeder cells, controlling the density of feeder cells, and passaging by manually mechanical cutting. As a result, hESCs retained a stable normal karyotype even after more than two years of cultivation under optimized conditions [6, 7]. In this study, we induced the malignant transformation of hESCs by continuous exposure to 0.001 *μ*g/ml MMC; subsequently, the cellular response was assessed. The data demonstrated that after the MMC treatment, the expression of *CTNNB1* and its target genes, especially proto-oncogenes *CCND1*, *C-MYC*, *MDM2*, and *BAX*, was upregulated, while that of the apoptosis-related molecule, *P21*, was downregulated. Moreover, the overexpression of *CTNNB1* accelerated the cell transition from G0/G1 to S phase. Conversely, *CTNNB1* depletion induced the G2/M cell cycle checkpoint arrest after MMC treatment. *CCND1* is a crucial downstream target gene of the Wnt/*β*-catenin signaling pathway, which coordinates the cell cycle progression [36, 37]. *CCND1* dysregulation leads to uncontrolled cell cycle progression, and its overexpression is correlated to genomic instability, rapid cell growth, cell bypass of critical cellular checkpoints, and neoplastic growth [37, 38]. In addition, pathologically active *MYC* and *MDM2* genes drive an abnormally rapid cell cycle to dysregulate cell proliferation [39]. *BAX* is a proapoptotic gene and might affect cell proliferation by directly modulating the cell cycle inhibitor *P21* [40, 41]. Thus, our results demonstrated that elevated expression of *CTNNB1* targets *CCND1* upregulation and *P21* downregulation, which in turn accelerates the transition of chromosomally unstable cells from G0/G1 to S phase. Conversely, *CTNNB1* depleted in hESCs induced G2/M phase arrest and survival crisis after MMC treatment.

Telomere is a conserved DNA-protein complex that maintains the stability of the chromosomes. Telomeres lose about 200 nucleotides per cell division. When a telomere degradation reaches a threshold level, uncapped telomeres activate cell senescence or apoptosis by enforcing the cell cycle arrest [18, 42]. Telomere dysfunction promotes tumorigenesis by inducing chromosomal instability in tumor-initiating cells, bypassing the cell cycle checkpoint, and increasing the proliferative competition of transformed cells [43, 44]. As reported, h*TERT* promoter mutations and *CTNNB1* mutations are significantly associated, and telomere and *CTNNB1* have a cooperative effect in tumorigenesis [25]. In this study, the MMC treatment elevated the number of telomere BFB cycles in *CTNNB1*-depleted cells and significantly reduced the proliferation and survival of *CTNNB1*-depleted cells within 7 days. This might be due to the mismatched end-to-end chromosome fusions and recombination effectuated by uncapped chromosomes leading to mitotic arrest and affecting the cellular fate in precrisis [45, 46]. Conversely, cells with MMC-induced overexpression of *CTNNB1* overcomes the survival crisis and contributed to uncontrolled proliferation. Moreover, *CTNNB1* maintains the telomere function by regulating the telomerase activity. Taken together, the current results indicated that *CTNNB1* silencing may exacerbate telomere deprotection, thereby increasing the sensitivity of cells to mutagens and promoting cell death during cell cycle arrest. Typically, the overexpression of *CTNNB1* conferred a proliferative advantage, culture adaptability, and resistance to apoptosis on chromosomally unstable cells by mediating telomere maintenance, and therefore driving the malignant transformation of hESCs.

**Figure 6:**
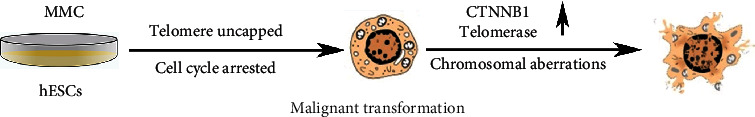
Malignant transformation of hESCs.

In summary, we proposed that *CTNNB1* is a key factor in the early stage of malignant transformation of hESCs by regulating telomere maintenance and promoting the proliferation of chromosomally unstable cells (Figure 6). Our data offered new insights into the complex etiology of malignant transformation of ESCs, and CTNNB1 serves as a potential prognostic safety biomarker for the application of hESCs in regenerative medicine and therapeutic target in the malignant transformation of stem cells.

## Data Availability

The data used to support the findings of this study are available from the corresponding author upon request.

## Supplementary Materials

Supplementary Materials Supplementary Figure 1: (a) Cell proliferation analysis by EdU labeling was performed in stably transfected sh*CTNNB1* groups, stably transfected empty vector group (MOCK), and uninfected control group (WT), respectively (mean ± SD, n =3; ∗*p* < 0.05*vs*. WT). (b) The G-band analysis after a long-term culture showed that shCTNNB1 hESCs retain a normal karyotype. Supplementary Figure 2: Expression of pluripotency markers in sh*CTNNB1* cells. Cellular morphology of hESCs (scale bar =100 *μ*m). Alkaline phosphatase (AKP) activity (scale bar =200 *μ*m) and the expression of TRA-1-60, TRA-1-81, SSEA-3, SSEA-4, SSEA-1, and NANOG were detected by immunofluorescence staining in sh*CTNNB1* hESCs. Nuclei were stained with DAPI (blue; insets) (scale bar =100 *μ*m). Supplementary Table 1. Primary antibodies used for immunofluorescence staining and Western blot analysis. Supplementary Table 2. Primers for RT-PCR and RT-qPCR.

## References

[1] Thomson J. A., Itskovitz-Eldor J., Shapiro S. S. (1998). Embryonic stem cell lines derived from human blastocysts. *Science*.

[2] Rebuzzini P., Zuccotti M., Redi C. A., Garagna S. (2016). Chromosomal abnormalities in embryonic and somatic stem cells. *Cytogenetic and Genome Research*.

[3] Rebuzzini P., Zuccotti M., Redi C. A., Garagna S. (2016). Achilles’ heel of pluripotent stem cells: genetic, genomic and epigenetic variations during prolonged culture. *Cellular and Molecular Life Sciences*.

[4] Kermi C., Aze A., Maiorano D. (2019). Preserving Genome Integrity During the Early Embryonic DNA Replication Cycles. *Genes*.

[5] Yang S., Lin G., Tan Y. Q. (2008). Tumor progression of culture-adapted human embryonic stem cells during long-term culture. *Genes, Chromosomes & Cancer*.

[6] Sun Y., Yang Y., Zeng S., Tan Y., Lu G., Lin G. (2014). Identification of proteins related to epigenetic regulation in the malignant transformation of aberrant karyotypic human embryonic stem cells by quantitative proteomics. *PLoS One*.

[7] Zhou D., Lin G., Zeng S. C. (2015). Trace levels of mitomycin C disrupt genomic integrity and lead to DNA damage response defect in long-term-cultured human embryonic stem cells. *Archives of Toxicology*.

[8] Liu Z., Guo J., Wang Y. (2017). CFTR- *β*-catenin interaction regulates mouse embryonic stem cell differentiation and embryonic development. *Cell Death and Differentiation*.

[9] Steinhart Z., Angers S. (2018). Wnt signaling in development and tissue homeostasis. *Development*.

[10] Majidinia M., Aghazadeh J., Jahanban-Esfahlani R., Yousefi B. (2018). The roles of Wnt/*β*-catenin pathway in tissue development and regenerative medicine. *Journal of Cellular Physiology*.

[11] Shang S., Hua F., Hu Z. W. (2017). The regulation of *β*-catenin activity and function in cancer: therapeutic opportunities. *Oncotarget*.

[12] Pan B. L., Wu L., Pan L. (2018). Up-regulation of microRNA-340 promotes osteosarcoma cell apoptosis while suppressing proliferation, migration, and invasion by inactivating the CTNNB1-mediated Notch signaling pathway. *Bioscience Reports*.

[13] Chang Y. H., Chu T. Y., Ding D. C. (2017). WNT/*β*-Catenin signaling pathway regulates non-tumorigenesis of human embryonic stem cells co-cultured with human umbilical cord mesenchymal stem cells. *Scientific Reports*.

[14] Bernal A., Tusell L. (2018). Telomeres: Implications for Cancer Development. *International Journal of Molecular Sciences*.

[15] Li F., Ge Y., Liu D., Songyang Z. (2020). The role of telomere-binding modulators in pluripotent stem cells. *Protein & Cell*.

[16] Chen X., Tang W. J., Shi J. B., Liu M. M., Liu X. H. (2020). Therapeutic strategies for targeting telomerase in cancer. *Medicinal Research Reviews*.

[17] Shay J. W. (2016). Role of telomeres and telomerase in aging and cancer. *Cancer Discovery*.

[18] De Vitis M., Berardinelli F., Sgura A. (2018). Telomere length maintenance in cancer: at the crossroad between telomerase and alternative lengthening of telomeres (ALT). *International Journal of Molecular Sciences*.

[19] Yuan X., Dai M., Xu D. (2020). Telomere-related markers for cancer. *Current Topics in Medicinal Chemistry*.

[20] Zeng S., Liu L., Sun Y. (2014). Telomerase-mediated telomere elongation from human blastocysts to embryonic stem cells. *Journal of Cell Science*.

[21] Bhargava R., Fischer M., O’Sullivan R. J. (2020). Genome rearrangements associated with aberrant telomere maintenance. *Current Opinion in Genetics & Development*.

[22] Turner K. J., Vasu V., Griffin D. K. (2019). Telomere biology and human phenotype. *Cells*.

[23] Graham M. K., Meeker A. (2017). Telomeres and telomerase in prostate cancer development and therapy. *Nature Reviews Urology*.

[24] Laberthonniere C., Magdinier F., Robin J. D. (2019). Bring it to an end: does telomeres size matter?. *Cells*.

[25] Zucman-Rossi J., Villanueva A., Nault J. C., Llovet J. M. (2015). Genetic landscape and biomarkers of hepatocellular carcinoma. *Gastroenterology*.

[26] Newell F., Kong Y., Wilmott J. S. (2019). Whole-genome landscape of mucosal melanoma reveals diverse drivers and therapeutic targets. *Nat Commun*.

[27] Lin G., Xie Y., OuYang Q. (2009). HLA-matching potential of an established human embryonic stem cell bank in China. *Cell Stem Cell*.

[28] Andrews P. W., Damjanov I., Simon D. (1984). Pluripotent embryonal carcinoma clones derived from the human teratocarcinoma cell line Tera-2. Differentiation in vivo and in vitro. *Laboratory Investigation*.

[29] Yang S., Lin G., Deng L., Lu G. X. (2012). Tumourigenic characteristics of embryonal carcinoma cells as a model for studying tumour progression of human embryonic stem cells. *Cell Proliferation*.

[30] Livak K. J., Schmittgen T. D. (2001). Analysis of relative gene expression data using real-time quantitative PCR and the 2(-Delta Delta C(T)) method. *Methods*.

[31] Ourliac-Garnier I., Londono-Vallejo A. (2017). Telomere length analysis by quantitative fluorescent in situ hybridization (Q-FISH). *Methods in Molecular Biology*.

[32] Xu Z., Robitaille A. M., Berndt J. D. (2016). Wnt/*β*-catenin signaling promotes self-renewal and inhibits the primed state transition in naïve human embryonic stem cells. *Proceedings of the National Academy of Sciences of the United States of America*.

[33] Henry M. P., Hawkins J. R., Boyle J., Bridger J. M. (2019). The genomic health of human pluripotent stem cells: genomic instability and the consequences on nuclear organization. *Frontiers in Genetics*.

[34] van de Moosdijk A. A. A., van de Grift Y. B. C., de Man S. M. A., Zeeman A. L., van Amerongen R. (2020). A novel Axin2 knock-in mouse model for visualization and lineage tracing of WNT/CTNNB1 responsive cells. *Genesis*.

[35] van Schie E. H., van Amerongen R. (2020). Aberrant WNT/CTNNB1 signaling as a therapeutic target in human breast cancer: weighing the evidence. *Frontiers in Cell and Development Biology*.

[36] Bisso A., Filipuzzi M., Gamarra Figueroa G. P. (2020). Cooperation between MYC and *β*‐Catenin in liver tumorigenesis requires Yap/Taz. *Hepatology*.

[37] Wang Q., He G., Hou M. (2018). Cell Cycle Regulation by Alternative Polyadenylation of CCND1. *Scientific Reports*.

[38] Katoh M. (2018). Multi-layered prevention and treatment of chronic inflammation, organ fibrosis and cancer associated with canonical WNT/*β*-catenin signaling activation (review). *International Journal of Molecular Medicine*.

[39] Scholz B. A., Sumida N., de Lima C. D. M. (2019). WNT signaling and AHCTF1 promote oncogenic MYC expression through super-enhancer-mediated gene gating. *Nature Genetics*.

[40] Brayer S., Joannes A., Jaillet M. (2017). The pro-apoptotic BAX protein influences cell growth and differentiation from the nucleus in healthy interphasic cells. *Cell Cycle*.

[41] Karimian A., Ahmadi Y., Yousefi B. (2016). Multiple functions of p21 in cell cycle, apoptosis and transcriptional regulation after DNA damage. *DNA Repair*.

[42] Hayashi M. T., Cesare A. J., Rivera T., Karlseder J. (2015). Cell death during crisis is mediated by mitotic telomere deprotection. *Nature*.

[43] Gunes C., Avila A. I., Rudolph K. L. (2018). Telomeres in cancer. *Differentiation*.

[44] Perera O. N., Sobinoff A. P., Teber E. T. (2019). Telomerase promotes formation of a telomere protective complex in cancer cells. *Science Advances*.

[45] Maciejowski J., Li Y., Bosco N., Campbell P. J., de Lange T. (2015). Chromothripsis and kataegis induced by telomere crisis. *Cell*.

[46] Feijoo P., Dominguez D., Tusell L., Genesca A. (2014). Telomere-dependent genomic integrity: evolution of the fusion-bridge-breakage cycle concept. *Current Pharmaceutical Design*.

