# Supplementary material for: Essentiality of CTNNB1 in Malignant Transformation of Human Embryonic Stem Cells under Long-Term Suboptimal Conditions
Source: Stem Cells Int. 2020 Sep 24;2020:5823676. doi: 10.1155/2020/5823676 (PMC7532415; doi:10.1155/2020/5823676)
Supplement: Supplementary Materials — Supplementary Figure 1: (a) Cell proliferation analysis by EdU labeling was performed in stably transfected shCTNNB1 groups, stably transfected empty vector group (MOCK), and uninfected control group (WT), respectively (mean ± SD, n =3; ∗p < 0.05vs. WT). (b) The G-band analysis after a long-term culture showed that shCTNNB1 hESCs retain a normal karyotype. Supplementary Figure 2: Expression of pluripotency markers in shCTNNB1 cells. Cellular morphology of hESCs (scale bar =100 μm). Alkaline phosphatase (AKP) activity (scale bar =200 μm) and the expression of TRA-1-60, TRA-1-81, SSEA-3, SSEA-4, SSEA-1, and NANOG were detected by immunofluorescence staining in shCTNNB1 hESCs. Nuclei were stained with DAPI (blue; insets) (scale bar =100 μm). Supplementary Table 1. Primary antibodies used for immunofluorescence staining and Western blot analysis. Supplementary Table 2. Primers for RT-PCR and RT-qPCR. [file 5823676.f1.docx]

**Essentiality of *CTNNB1* in Malignant Transformation of Human Embryonic Stem Cells under Long-term Suboptimal Conditions**

Jie Liu,^1, *^ Sicong Zeng,^1, 3, *^ Yang Wang,^1^ Juan Yu,^2^ Qi Ouyang,^1, 2^ Liang Hu,^1, 2^ Di Zhou,^1, 2^ Ge Lin,^1, 2^ and Yi Sun^1, 2, 3­^

^1^ Institute of Reproductive & Stem Cell Engineering, School of Basic Medical Science, Central South University, Changsha 410001, China

^2^ National Engineering and Research Center of Human Stem Cells, Changsha 410001, China

^3^ Key laboratory of Stem Cells and Reproductive Engineering, Ministry of Health, Changsha 410001, China

^*^These authors contributed equally to this work.

Correspondence should be addressed to Yi Sun; sunyi66@csu.edu.cn

Supplementary Figures





Supplementary Figure 1: (a) Cell proliferation analysis by EdU labeling was performed in stably transfected sh*CTNNB1* groups, stably transfected empty vector group (MOCK), and uninfected control group (WT), respectively (mean ± SD, n = 3; *p<0.05 vs. WT). (b) The G-band analysis after a long-term culture showed that the sh-*CTNNB1* hESCs retained a normal karyotype.


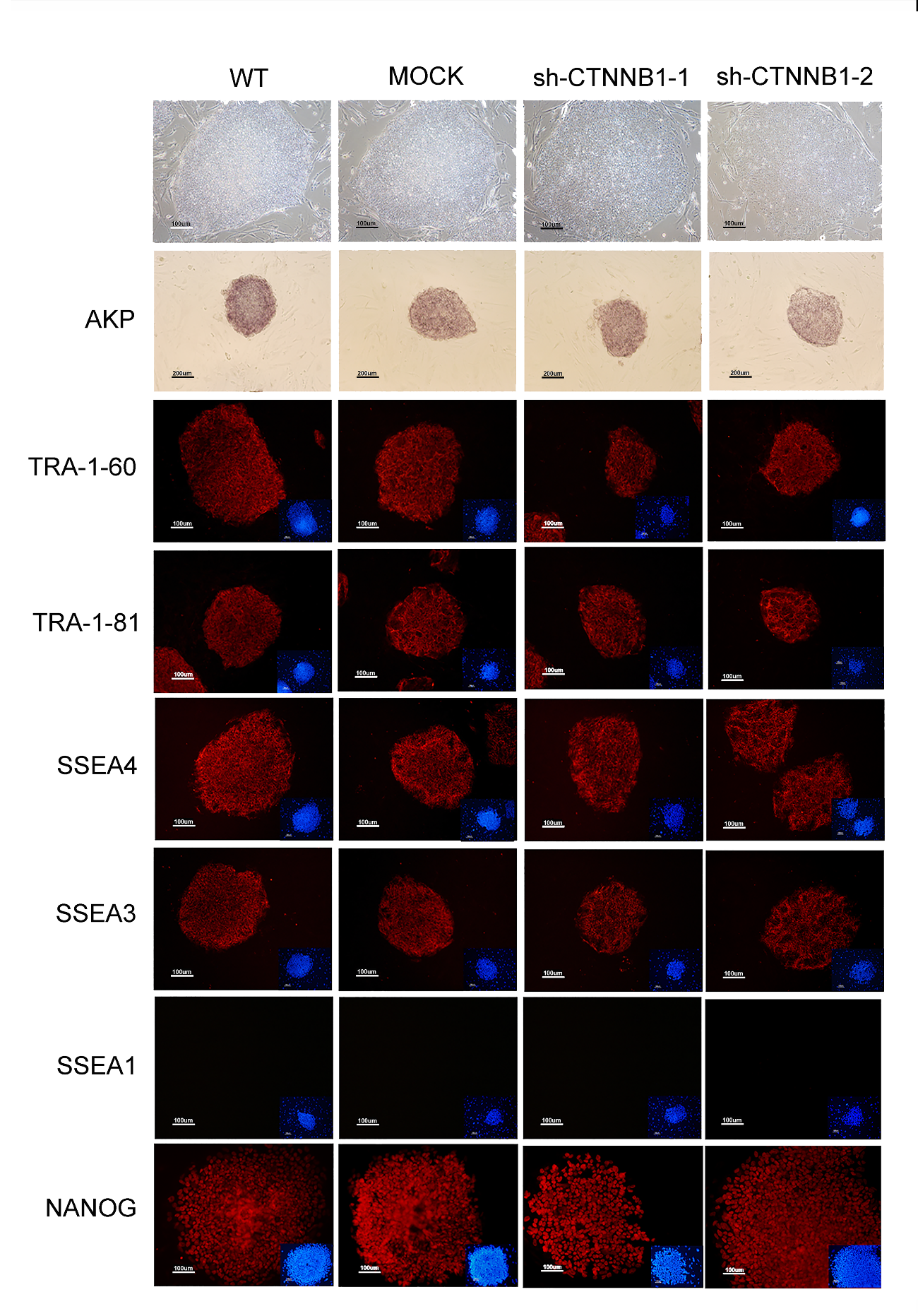


Supplementary Figure 2: Expression of pluripotency markers in sh*CTNNB1* cells. Cellular morphology of hESCs (scale bar = 100 μm). Alkaline phosphatase (AKP) activity (scale bar = 200 μm) and the expression of TRA-1-60, TRA-1-81, SSEA-3, SSEA-4, SSEA-1, and NANOG were detected by immunofluorescence staining in sh*CTNNB1* hESCs. Nuclei were stained with DAPI (blue; insets) (scale bar = 100 μm).

Supplementary Tables

Supplementary Table 1. Primary antibodies used for immunofluorescence staining and Western blot analysis

| **Protein** | **Host** | **Vendor** |
| --- | --- | --- |
| OCT4 | Mouse monoclonal | Santa Cruz Biotechnology |
| TRA-1-60 | Mouse monoclonal | Chemicon |
| TRA-1-81 | Mouse monoclonal | Chemicon |
| SSEA-3 | Rat monoclonal | Santa Cruz Biotechnology |
| SSEA-4 | Mouse monoclonal | R&D Systems |
| SSEA-1 | Mouse monoclonal | R&D Systems |
| NANOG | Mouse monoclonal | Abcam |
| CTNNB1 | Rabbit monoclonal | Sigma |
| β-Actin | Mouse monoclonal | Sigma |
| β-Tubulin | Mouse monoclonal | Sigma |
| SMA | Mouse monoclonal | Millipore |
| AFP | Mouse monoclonal | Sigma |

Supplementary Table 2. Primers for RT-PCR and RT-qPCR

| *OCT4* | Forward Sequence: | 5’-AGCGAACCAGTATCGAGAAC-3’ |
| --- | --- | --- |
|  | Reverse Sequence: | 5’-TTACAGAACCACACTCGGAC-3’ |
| *NANOG* | Forward Sequence: | 5’-TGAACCTCAGCTACAAACAG-3’ |
|  | Reverse Sequence: | 5’-TGGTGGTAGGAAGAGTAAAG-3’ |
| *TDGF1* | Forward Sequence: | 5’-TCCTTCTACGGACGGAACTG-3’ |
|  | Reverse Sequence: | 5’-AGAAATGCCTGAGGAAAGCA-3’ |
| *TERF1* | Forward Sequence: | 5’-ACAGCGCCGAGGCTATTATT-3’ |
|  | Reverse Sequence: | 5’-GTGTAATACGCTCATCAACT-3’ |
| *KLF4* | Forward Sequence: | 5’-TCTCAAGGCACACCTGCGAA-3’ |
|  | Reverse Sequence: | 5’-TAGTGCCTGGTCAGTTCATC-3’ |
| *REX1* | Forward Sequence: | 5’-TGAAAGCCCACATCCTAACG-3’ |
|  | Reverse Sequence: | 5’-CAAGCTATCCTCCTGCTTTGG-3’ |
| *LEFTYA* | Forward Sequence: | 5’-GGGAATTGGGATACCTGGAT-3’ |
|  | Reverse Sequence: | 5’-CTAAATATGCACGGGCAAGG-3’ |
| *CRIPTO* | Forward Sequence: | 5’-TCCTTCTACGGACGGAACTG-3’ |
|  | Reverse Sequence: | 5’-AGAAATGCCTGAGGAAAGCA-3’ |
| *THY-1* | Forward Sequence: | 5’-AGAATACCAGCAGTTCACCCATCC-3’ |
|  | Reverse Sequence: | 5’-CCTCACACTTGACCAGTTTGTCTCTG-3’ |
| *CTNNB1* | Forward Sequence: | 5’-CACAAGCAGAGTGCTGAAGGTG-3’ |
|  | Reverse Sequence: | 5’-GATTCCTGAGAGTCCAAAGACAG-3’ |
| *KRT17* | Forward Sequence: | 5’-GGTGGGTGGTGAGATCAATGT-3’ |
|  | Reverse Sequence: | 5’-CGCGGTTCAGTTCCTCTGTC-3’ |
| *ACTC1* | Forward Sequence: | 5’-CATCCTGACCCTGAAGTATCCCATC-3’ |
|  | Reverse Sequence: | 5’-CCCTCATAGATGGGGACATTGTGAG-3’ |
| *RUNX1* | Forward Sequence: | 5’-CTGCCCATCGCTTTCAAGGT-3’ |
|  | Reverse Sequence: | 5’-GCCGAGTAGTTTTCATCATTGCC-3’ |
| *HAND1* | Forward Sequence: | 5’-AAGAGAACCAGACGCAGGAA-3’ |
|  | Reverse Sequence: | 5’-GGCAGGATGAACAAACACCT-3’ |
| *GATA4* | Forward Sequence: | 5’-GTGTCCCAGACGTTCTCAGTC-3’ |
|  | Reverse Sequence: | 5’-GGGAGACGCATAGCCTTGT-3’ |
| *GATA6* | Forward Sequence: | 5’-GTGCCCAGACCACTTGCTAT-3’ |
|  | Reverse Sequence: | 5’-TGGAATTATTGCTATTACCAGAGC-3’ |
| *SOX17* | Forward Sequence: | 5’-GGCGCTGACACCAGACTT-3’ |
|  | Reverse Sequence: | 5’-TCTCGCCTCGTTTTGACTTT-3’ |
| *HCG* | Forward Sequence: | 5’-GTCAACACCACCATCTGTGC-3’ |
|  | Reverse Sequence: | 5’-GGCCTTTGAGGAAGAGGAGT-3’ |
| *CDX2* | Forward Sequence: | 5’-GGGCTC TCTGAGAGGCAGGT-3’ |
|  | Reverse Sequence: | 5’-CCTTTGCTCTGCGGTTCTG-3’ |
| *GAPDH* | Forward Sequence: | 5’-ACCACAGTCCATGCCATCAC-3’ |
|  | Reverse Sequence: | 5’-CCACCACCCTGTTGCTGTA-3’ |
| *C-MYC* | Forward Sequence: | 5’-AGCGACTCTGAGGAGGAACAA-3’ |
|  | Reverse Sequence: | 5’-GTGGGCTGTGAGGAGGTTTG-3’ |
| *BCL2* | Forward Sequence: | 5’-ATCGCCCTGTGGATGACTGAGT-3’ |
|  | Reverse Sequence: | 5’-GCCAGGAGAAATCAAACAGAGGC-3’ |
| *CCND1* | Forward Sequence: | 5’-TCTACACCGACAACTCCATCCG-3’ |
|  | Reverse Sequence: | 5’-TCTGGCATTTTGGAGAGGAAGTG-3’ |
| *P21* | Forward Sequence: | 5’-AGGTGGACCTGGAGACTCTCAG-3’ |
|  | Reverse Sequence: | 5’-TCCTCTTGGAGAAGATCAGCCG-3’ |
| *MDM2* | Forward Sequence: | 5’-TGTTTGGCGTGCCAAGCTTCTC-3’ |
|  | Reverse Sequence: | 5’-CACAGATGTACCTGAGTCCGATG-3’ |
| *BAX* | Forward Sequence: | 5’-TCAGGATGCGTCCACCAAGAAG-3’ |
|  | Reverse Sequence: | 5’-TGTGTCCACGGCGGCAATCATC-3’ |
| *SURVIVN* | Forward Sequence: | 5’-GCATGGGTGCCCCGACGTTG-3’ |
|  | Reverse Sequence: | 5’-GCTCCGGCCAGAGGCCTCAA-3’ |
